# Functional genomics–guided design of CAR-T and CAR-NK therapies in hematological malignancies: aligning cellular engineering with immune escape and microenvironmental resistance

**DOI:** 10.3389/fgeed.2026.1878460

**Published:** 2026-08-13

**Authors:** Shiqi Cai, Yang Tan, Qian Yang, Juan Huang

**Affiliations:** 1 Department of Hematology, Sichuan Academy of Medical Sciences and Sichuan People’s Hospital, Chengdu, China; 2 School of Life Science and Technology, University of Electronic Science and Technology of China, Chengdu, China; 3 Department of Hematology, The Affiliated Hospital of Southwest Medical University, Luzhou, China

**Keywords:** CAR-NK cell therapy, CAR-T cell therapy, genome editing, immune escape, tumor microenvironment

## Abstract

**Background:**

Chimeric antigen receptor T-cell therapy has changed the treatment landscape of relapsed or refractory hematological malignancies, but primary non-response and post-infusion relapse remain frequent clinical problems. In aggressive B-cell lymphomas, acute leukemias, and multiple myeloma, treatment failure is often driven by overlapping mechanisms rather than a single resistance pathway. These include antigen loss or reduced antigen density, impaired immune recognition, defective inflammatory signaling, checkpoint-mediated suppression, metabolic stress, and limited effector-cell persistence within suppressive disease niches.

**Main Body:**

Genome engineering has become an important tool for both identifying and addressing these resistance mechanisms. CRISPR-based functional screening, single-cell perturbation approaches, and multi-omics profiling allow immune escape and tumor microenvironment-mediated resistance to be defined more functionally, rather than inferred only from correlative datasets. These insights can inform the design of CAR-T and CAR-NK therapies through multi-target or logic-gated receptors, checkpoint or exhaustion-pathway editing, cytokine-supported and armored constructs, metabolic fitness enhancement, and selected multiplex-editing strategies. In parallel, CAR-NK cells, universal allogeneic CAR-T products, and stem-cell-derived platforms may provide additional options in relapse-prone or heavily pretreated patients, particularly when autologous T-cell fitness, manufacturing feasibility, or repeat dosing is a concern.

**Conclusion:**

A resistance-guided approach may help align engineered cellular therapy design with the dominant mechanisms of treatment failure in high-risk hematological malignancies. Rather than simply increasing engineering complexity, future CAR-T and CAR-NK development should link each modification to a measurable resistance mechanism, a feasible biomarker, and a clinically testable benefit. Prospective validation, genomic safety assessment, manufacturing consistency, and long-term monitoring will be essential before resistance-matched cellular immunotherapy can be broadly integrated into clinical practice.

## 1 Introduction

Adoptive cellular immunotherapy, particularly chimeric antigen receptor T (CAR-T) cell therapy, has reshaped the treatment landscape of hematological malignancies. In selected patients with B-cell leukemias, aggressive lymphomas, and multiple myeloma, engineered immune cells can induce deep and durable remissions, establishing cellular therapy as an important treatment modality alongside chemotherapy, targeted agents, and stem cell transplantation (Neelapu et al., 2018; Majzner and Mackall, 2019; Peng et al., 2024). Emerging CAR-NK cells and universal off-the-shelf platforms have further expanded the therapeutic possibilities of cell-based immunotherapy. These platforms broaden the clinical reach of cellular immunotherapy by reducing dependence on patient-specific autologous manufacturing, shortening treatment availability, and enabling repeat or staged dosing when rapid disease control is required. CAR-NK cells also combine CAR-directed specificity with innate stress-ligand and missing-self recognition, potentially offering activity in antigen-heterogeneous disease with a lower risk of severe cytokine release syndrome, neurotoxicity, and graft-versus-host disease. Universal allogeneic CAR-T and stem-cell-derived immune products may provide more standardized starting material and support multiplex resistance-countering edits before large-scale manufacturing. These features are particularly relevant for heavily pretreated patients, individuals with poor autologous T-cell fitness, and patients with rapidly progressive disease who may not tolerate the delay associated with individualized CAR-T production. (Khawar and Sun, 2021). However, primary non-response or relapse remains common, with 40%–60% of patients in aggressive B-cell lymphomas progressing within 6–12 months post-CAR-T infusion (Aymard et al., 2026; Erbella et al., 2026), underscoring persistent biological barriers to durable efficacy.

Two interconnected mechanisms drive treatment failure: immune escape and tumor microenvironment (TME)–mediated resistance. Immune escape arises from antigen loss/downregulation, defects in antigen processing/presentation, interferon signaling disruption, and activation of tumor survival pathways, enabling adaptive genetic/epigenetic evolution under immune pressure (Wang M. R. et al., 2026; Lei et al., 2024). Concurrently, the TME imposes multilayered suppression via checkpoints, inhibitory cytokines, myeloid suppressor cells, stromal/ECM barriers, and metabolic constraints, promoting effector cell exhaustion and limited persistence (Peng et al., 2024; Sterner and Sterner, 2021). These barriers are amplified in high-risk settings, including genetically unstable, heavily pretreated, or immunosuppressed tumors.

Genome engineering, especially CRISPR-based approaches, has provided a powerful way to dissect and potentially counter these resistance mechanisms. Genome-wide loss- and gain-of-function screens, *in vivo* perturbation models, single-cell transcriptomic readouts, and combinatorial editing have helped identify regulators of antigen presentation, interferon response, apoptosis, checkpoint signaling, and effector-cell fitness (Peng et al., 2024; Beatriz Coutinho De Oliveira et al., 2025). These tools allow resistance programs to be studied functionally, rather than only inferred from correlative genomic or transcriptomic data. In parallel, multiplex editing can be used to modify CAR-T and CAR-NK cells by enhancing persistence, reducing suppressive signaling, rewiring cytokine pathways, or incorporating safety-control elements. In selected contexts, genome editing may also support bidirectional strategies that target both engineered effector cells and resistance-promoting tumor or microenvironmental components (Wang Y. et al., 2026).

This dual role makes genome engineering both a discovery platform and a therapeutic design tool. By linking resistance mechanisms with corresponding engineering strategies, functional genomics can help guide the rational design of cellular products for high-risk hematologic malignancies. This is especially important when antigen heterogeneity, impaired immune recognition, T-cell exhaustion, and microenvironmental suppression coexist, making single-target or potency-focused approaches insufficient. A mechanism-matched engineering strategy therefore provides a practical framework for aligning specific resistance pathways with tailored CAR-T or CAR-NK modifications (Balkhi et al., 2025).

In this review, we discuss how genome engineering and functional genomics can be integrated with CAR-T and CAR-NK design to address immune escape and tumor microenvironment-mediated resistance in hematological malignancies. We first summarize genome editing as a discovery platform for resistance mechanisms. We then map major tumor-intrinsic and microenvironmental resistance pathways to corresponding engineering countermeasures. Next, we evaluate universal, CAR-NK, and other alternative cellular platforms in escape-prone disease settings. Finally, we propose a mechanism-guided framework for high-risk disease and discuss the translational challenges that must be addressed before resistance-matched cellular immunotherapy can be broadly implemented.

## 2 Genome engineering as a discovery engine for immune escape and therapeutic resistance

### 2.1 CRISPR functional screening identifies core drivers of immune escape

Programmable genome engineering-particularly CRISPR-based loss- and gain-of-function screening-has revolutionized the unbiased discovery of genetic determinants governing tumor resistance to immune-mediated killing. Unlike correlative genomic analyses, these functional screens directly test causality under immune selection pressure, enabling genome-scale identification of convergent resistance modules (Shifrut et al., 2018).

Across tumor models exposed to cytotoxic T or NK cells, interferon-γ stimulation, or checkpoint blockade, CRISPR screens consistently highlight disruptions in IFN-γ–JAK–STAT signaling, antigen processing and presentation machinery (including β2-microglobulin and TAP components), and programmed cell death pathways (Patel et al., 2017; Manguso et al., 2017). Loss-of-function alterations in JAK1 or JAK2 impair interferon responsiveness, reduce MHC-I expression and chemokine induction, and diminish immune recognition (Garcia-Diaz et al., 2017). Both *in vitro* and *in vivo* CRISPR screening studies—including platforms applied in CAR-T–relevant contexts—have validated these pathways as clinically relevant escape routes, with resistant tumor clones frequently enriched for antigen presentation or interferon signaling defects after immunotherapy exposure (Pan et al., 2018; Lawson et al., 2020). Additional hits, including metabolic regulators and signaling adaptors, further underscore the polygenic and partially redundant nature of immune escape, which often involves parallel pathway compensation rather than single-gene events (Pech et al., 2019).

Together, these findings provide a functional resistance blueprint: by defining recurrent failure nodes such as interferon signaling and antigen presentation axes, CRISPR screening directly informs resistance-matched CAR-T and CAR-NK engineering strategies designed to anticipate or counter dominant escape mechanisms (Lei et al., 2024).

### 2.2 Single-cell perturbation genomics reveals heterogeneous resistance states

While bulk functional screens identify core resistance genes, immune escape typically manifests in a heterogeneous and state-dependent manner driven by genetic, epigenetic, and microenvironmental variability. Integration of CRISPR perturbations with single-cell transcriptomic readouts—including Perturb-seq and CROP-seq platforms—enables high-resolution mapping of how specific gene edits reshape immune-relevant cellular states across tumor subpopulations (Adamson et al., 2016; Dixit et al., 2016).

These studies demonstrate that perturbation of the same pathway—such as interferon response regulators—can produce divergent transcriptional outcomes depending on chromatin context, lineage identity, and baseline cellular state (Frangieh et al., 2021). Disruption of antigen presentation pathways, for example, may trigger compensatory regulatory circuits and adaptive transcriptional reprogramming, supporting a network-based model in which immune escape emerges from dynamic regulatory rewiring rather than linear pathway shutdown (Burr et al., 2019). Recent *in vivo* single-cell CRISPR approaches further reveal heterogeneous evolutionary programs under immune pressure, including distinct cytokine-dependent clonal expansion trajectories (Renz et al., 2024).

This functional heterogeneity helps explain the limited durability of mono-mechanistic interventions in high-risk hematological malignancies and supports the use of multiplex and logic-based engineering strategies, such as multi-target CAR constructs and combinatorial signaling rewiring (Balkhi et al., 2025). Single-cell perturbation maps also provide candidate biomarkers for dominant resistance programs, potentially enabling more personalized engineered cell therapy selection. In this way, perturbation genomics functions as a state-mapping platform that links gene function to phenotypic diversity and guides the design of more robust engineered immune cells.

### 2.3 Genome editing enables mechanism-driven models of high-risk and escape-prone disease

Genome editing extends beyond discovery screening to enable construction of mechanistically faithful preclinical models that recapitulate high-risk and immune-evasive disease features. Combinatorial CRISPR editing can introduce oncogenic drivers, tumor suppressor losses, and genomic instability–associated alterations, generating heterogeneous and immune-resistant model systems that more closely resemble aggressive leukemias and lymphomas (Stadtmauer et al., 2020).

These engineered models more accurately simulate evolutionary trajectories under immune pressure than single-mutation systems, enabling systematic analysis of how mutation constellations influence antigenicity, cytokine responsiveness, and susceptibility to immune-mediated killing (Adamson et al., 2016). Parallel editing of immune compartments—including checkpoint and cytokine receptor pathways in T or NK cells—supports bidirectional experimental designs in which engineered effectors are tested against genetically defined tumor targets (Wang Y. et al., 2026).

Integration with patient-derived xenografts, three-dimensional organoids, and immune co-culture systems further enhances translational relevance. Edited organoid–immune platforms allow functional evaluation of antigen heterogeneity, stromal suppression, and resistance-countering strategies in clinically proximal settings (Neal et al., 2018). Recent *in vivo* CRISPR applications in xenograft models of leukemia and lymphoma have validated these bidirectional systems for testing multiplex editing strategies and persistence-enhancing modifications (Li et al., 2023).

**FIGURE 1:**
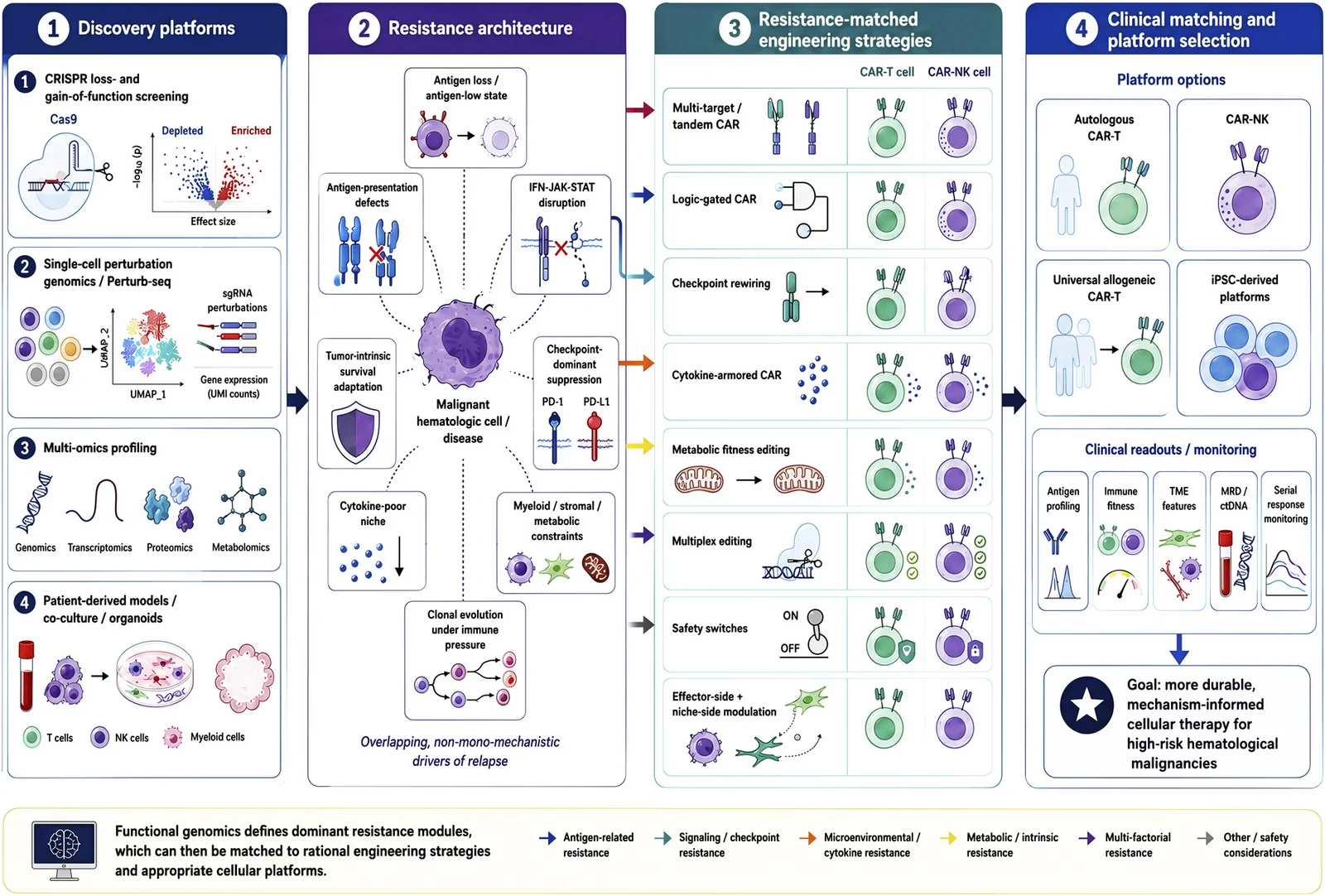
Schematic overview of the resistance-oriented workflow to design optimized CAR-T and CAR-NK therapies for high-risk hematologic malignancies.

This resistance-guided logic is summarized in Figure 1. Functional genomics and multi-omics profiling first define dominant resistance modules, which are then linked to corresponding CAR-T or CAR-NK engineering strategies and finally integrated with platform selection and clinical monitoring. This framework emphasizes that engineered cellular therapy should not be optimized solely for maximal potency, but should be matched to the specific mechanisms of immune escape and microenvironmental resistance present in high-risk hematological malignancies (Peng et al., 2024).

### 2.4 Applicability and limitations across tumor contexts

Many functional-genomic studies discussed in this review were initially performed in solid-tumor systems. Although several resistance pathways, including antigen escape, interferon-signaling disruption, checkpoint activation, cytokine limitation, and metabolic adaptation, appear broadly conserved across cancer types (Lawson et al., 2020), important biological differences exist between solid tumors and hematologic malignancies. Hematologic cancers primarily reside within bone marrow and lymphoid niches characterized by distinct stromal interactions, immune-cell trafficking patterns, antigen accessibility, and therapeutic exposure profiles (Sterner and Sterner, 2021; Yang et al., 2024). Therefore, findings derived from solid-tumor models should be interpreted as mechanistic hypotheses that inform resistance biology, rather than direct evidence for hematologic malignancies, and require validation within disease-specific hematologic contexts whenever possible.

Examples of disease-specific resistance mechanisms include antigen escape following CD19-directed CAR-T therapy in DLBCL, cytokine-dependent immune suppression within AML bone marrow niches, and BCMA modulation in multiple myeloma. These examples highlight how common resistance themes may manifest differently across hematologic malignancies and underscore the need for disease-specific validation. Nevertheless, the conserved biological processes identified through functional-genomic studies provide a useful conceptual foundation for resistance-matched engineering strategies.

## 3 Immune escape mechanisms and resistance-matched engineering in CAR-T and CAR-NK therapies

Immune escape remains a major barrier to durable responses in CAR-T and CAR-NK therapies for hematological malignancies. Relapse analyses and functional genomic studies have implicated antigen alteration, impaired immune signaling, and tumor-intrinsic survival adaptation as recurrent contributors to treatment failure (Majzner and Mackall, 2019; Aymard et al., 2026). However, these mechanisms rarely operate as isolated events. In post–cell therapy relapse, antigen instability, lineage plasticity, impaired inflammatory signaling, and selection of apoptosis-resistant clones may coexist within the same disease course. This has shifted cellular therapy design away from simply increasing effector potency and toward strategies that match defined resistance mechanisms with appropriate CAR architecture, editing modules, and cellular platforms (Lei et al., 2024). This section summarizes major tumor-intrinsic escape pathways and their corresponding engineering countermeasures in CAR-T and CAR-NK systems.

### 3.1 Antigen loss and modulation: multi-target and logic-gated CAR designs

Antigen escape, including complete antigen loss, reduced surface density, epitope alteration, or lineage switch, is a well-recognized mechanism of relapse after antigen-directed cellular therapy (Balkhi et al., 2025; Neelapu et al., 2017). This problem is particularly relevant in CD19-directed therapy for B-cell malignancies and BCMA-directed therapy for multiple myeloma, where selective pressure can favor antigen-low or antigen-negative clones. Functional CRISPR screens and relapse profiling studies have also linked antigen modulation to altered antigen processing, transcriptional silencing, and broader adaptive reprogramming (Patel et al., 2017).

Multi-target strategies attempt to reduce the risk of single-antigen escape by broadening tumor recognition. These include dual or bicistronic CARs, tandem CAR constructs, and combinatorial receptor systems that recognize more than one tumor-associated antigen (Wang M. R. et al., 2026; Roybal et al., 2016). AND/OR logic configurations may improve selectivity and reduce dependence on a single antigen, while synNotch-based receptor cascades can couple primary antigen recognition to inducible secondary CAR expression, allowing more context-dependent targeting (Morsut et al., 2016).

However, multi-antigen targeting should not be viewed as a complete solution to relapse. Antigen loss often occurs together with poor effector expansion, lineage plasticity, or suppressive microenvironmental signals. Therefore, antigen-pair selection needs to move beyond empirical combinations and should ideally be informed by co-retention, co-expression stability, and disease-specific dependency patterns. Functional genomic screening may help identify antigen combinations that are less vulnerable to coordinated downregulation, especially in genomically unstable or high-risk disease settings (Stadtmauer et al., 2020). Logic-gated CAR-NK platforms represent one developing example of this approach, combining multi-antigen recognition with innate immune sensing and inhibitory gate logic to improve tumor selectivity (Khawar and Sun, 2021).

Beyond conventional dual-target and tandem CAR constructs, modular CAR platforms have emerged as a flexible strategy for addressing antigen heterogeneity and adaptive immune escape. Systems such as SUPRA CAR, UniCAR, and RevCAR separate antigen recognition modules from intracellular signaling domains, enabling programmable target switching, sequential antigen targeting, and dynamic control of CAR activity (Cho et al., 2018; Bachmann, 2019; Liu et al., 2019; Sutherland et al., 2020). These modular architectures may reduce selective pressure on individual antigens and provide greater adaptability in diseases characterized by clonal evolution or fluctuating antigen expression. Although most modular CAR systems remain in early clinical development, they represent an important extension of resistance-matched engineering strategies.

### 3.2 Maintaining tumor recognition despite antigen presentation defects

Genome-wide functional screens repeatedly identify defects in MHC class I assembly and interferon response pathways as recurrent immune escape mechanisms (Garcia-Diaz et al., 2017; Pan et al., 2018). These abnormalities can reduce tumor immunogenicity and impair endogenous T-cell recognition. CAR-based recognition has an important advantage in this setting because it is not dependent on peptide-MHC presentation (Peng et al., 2024). This feature partly explains why CAR-T and CAR-NK therapies may remain active even when conventional T-cell recognition pathways are compromised.

Nevertheless, MHC-independent recognition does not eliminate all consequences of antigen-presentation defects. Interferon-pathway disruption may still reduce chemokine production, inflammatory amplification, and recruitment of endogenous immune cells, thereby weakening the broader antitumor response. For this reason, engineering strategies increasingly focus not only on bypassing MHC restriction but also on preserving stable surface targetability. Surfaceome-informed antigen discovery, supported by CRISPR and proteogenomic profiling, may help identify antigens that are more consistently retained across resistant tumor states (Manguso et al., 2017).

Additional strategies aim to stabilize or introduce targetable surface signals. Targeted knock-in of shared tumor-associated antigens or stress-induced ligands may enhance immune recognition in selected experimental settingss (Pech et al., 2019). Precision editing approaches, including base editing, can also modulate antigen expression with less broad genomic disruption than conventional nuclease-based approaches (Eyquem et al., 2017; Murray et al., 2025). At present, these approaches remain largely translational or preclinical, but they provide a rational direction for antigen-low or presentation-defective high-risk disease (Frangieh et al., 2021; Bartoszewska et al., 2024).

### 3.3 Engineering strategies to overcome tumor-intrinsic resistance

Tumor cells may also escape engineered immune-cell killing despite preserved antigen expression. This form of resistance can be driven by activation of anti-apoptotic pathways, stress-adaptation programs, altered inflammatory signaling, or reduced sensitivity to cytotoxic effector mechanisms (Lawson et al., 2020). Functional screens have identified apoptotic regulators and inflammatory signaling nodes that influence susceptibility to T-cell- or NK-cell-mediated killing (Pan et al., 2018). These findings are clinically relevant because relapse after CAR therapy may reflect not only loss of recognition but also survival of tumor clones that are intrinsically harder to eliminate.

Armored CAR designs provide one approach to this problem. These constructs can secrete cytokines or immune-modulating factors that enhance local effector-cell fitness, support persistence, and recruit endogenous immune responses (Yang et al., 2024; Carcopino et al., 2024). CAR-T and CAR-NK platforms incorporating cytokines such as IL-15 or IL-18 have shown improved expansion and functional activity in preclinical and early clinical studies (Huang et al., 2024; Yeku and Brentjens, 2016). Activation-dependent or locally restricted expression may help reduce systemic inflammatory toxicity while preserving activity within the tumor niche.

A complementary strategy is to combine engineered immune cells with agents that lower tumor survival thresholds. In lymphoma, leukemia, and myeloma, such combinations may include drugs targeting anti-apoptotic signaling, epigenetic regulation, or stress-response pathways, depending on the dominant resistance program. CRISPR-derived resistance maps can help prioritize these combinations by identifying pathways that directly modulate immune-killing sensitivity. This approach is most useful when it is used to guide rational pairing rather than simply adding more agents to intensify treatment.

### 3.4 Interferon signaling escape: cytokine pathway rewiring in effector cells

Disruption of interferon signaling, including alterations in JAK–STAT pathway components, has repeatedly emerged from functional screens as a mechanism of immune evasion (Patel et al., 2017; Garcia-Diaz et al., 2017). These defects can reduce chemokine induction, antigen-presentation support, and inflammatory feedback within the tumor environment. In CAR-based therapy, the consequences of interferon-pathway escape may be context-dependent. CAR recognition itself is MHC-independent, but interferon defects can still impair the local immune amplification that supports sustained antitumor activity.

Because restoring tumor interferon responsiveness is difficult, many engineering strategies focus on increasing effector-cell autonomy. Genome-edited CAR-T and CAR-NK cells can be modified to reduce dependence on suppressible cytokine axes through alternative survival signaling, synthetic cytokine support modules, or enhanced receptor signaling pathways (Alb et al., 2024). Editing inhibitory signaling nodes or strengthening pro-survival circuits may allow engineered cells to maintain function even when tumor-side inflammatory signaling is impaired (Kong et al., 2024). This strategy may be particularly relevant for CAR-NK platforms, whose innate recognition systems and signaling redundancy could provide additional resistance to tumor-side immune escape (Yang et al., 2024).

However, cytokine and signaling rewiring must be carefully balanced. Excessive cytokine support may increase inflammatory toxicity or drive abnormal effector-cell expansion, whereas insufficient support may fail to overcome hostile tumor niches (Yeku and Brentjens, 2016). Future designs will need to define the level and timing of cytokine signaling that improves persistence without compromising safety.

### 3.5 Multiplex and circuit-level engineering for polygenic escape

Functional genomic data suggest that immune escape is often polygenic, adaptive, and shaped by compensatory bypass pathways (Pan et al., 2018; Lawson et al., 2020). This helps explain why single-axis interventions may produce incomplete or short-lived benefit in high-risk hematological malignancies. Multiplex genome editing and circuit-level CAR engineering aim to address several resistance layers within the same engineered cell product.

Multiplex editing strategies may combine checkpoint modification, exhaustion resistance, metabolic support, cytokine signaling enhancement, or optimized CAR insertion (Garcia-Robledo et al., 2025). Circuit-level designs add another layer of control through inducible activation, feedback regulation, or logic-gated recognition (Li et al., 2022). Combinatorial CRISPR perturbation platforms can help identify edit combinations that produce synergistic improvements in engineered cell function rather than additive or redundant effects (Dixit et al., 2016).

Even so, more complex engineering does not automatically translate into better clinical performance. Each additional edit may introduce manufacturing challenges, genomic safety concerns, altered differentiation states, or unanticipated functional tradeoffs. Therefore, multiplex and circuit-based strategies should be prioritized for disease contexts in which resistance is demonstrably multilayered, such as antigen-heterogeneous, relapse-prone, or heavily pretreated malignancies (Jafarzadeh et al., 2025). In this setting, perturbation network maps may help distinguish rational edit combinations from excessive engineering complexity, supporting a more clinically grounded form of resistance-matched cellular therapy.

## 4 Tumor microenvironment resistance and bidirectional genome engineering strategies

**FIGURE 2:**
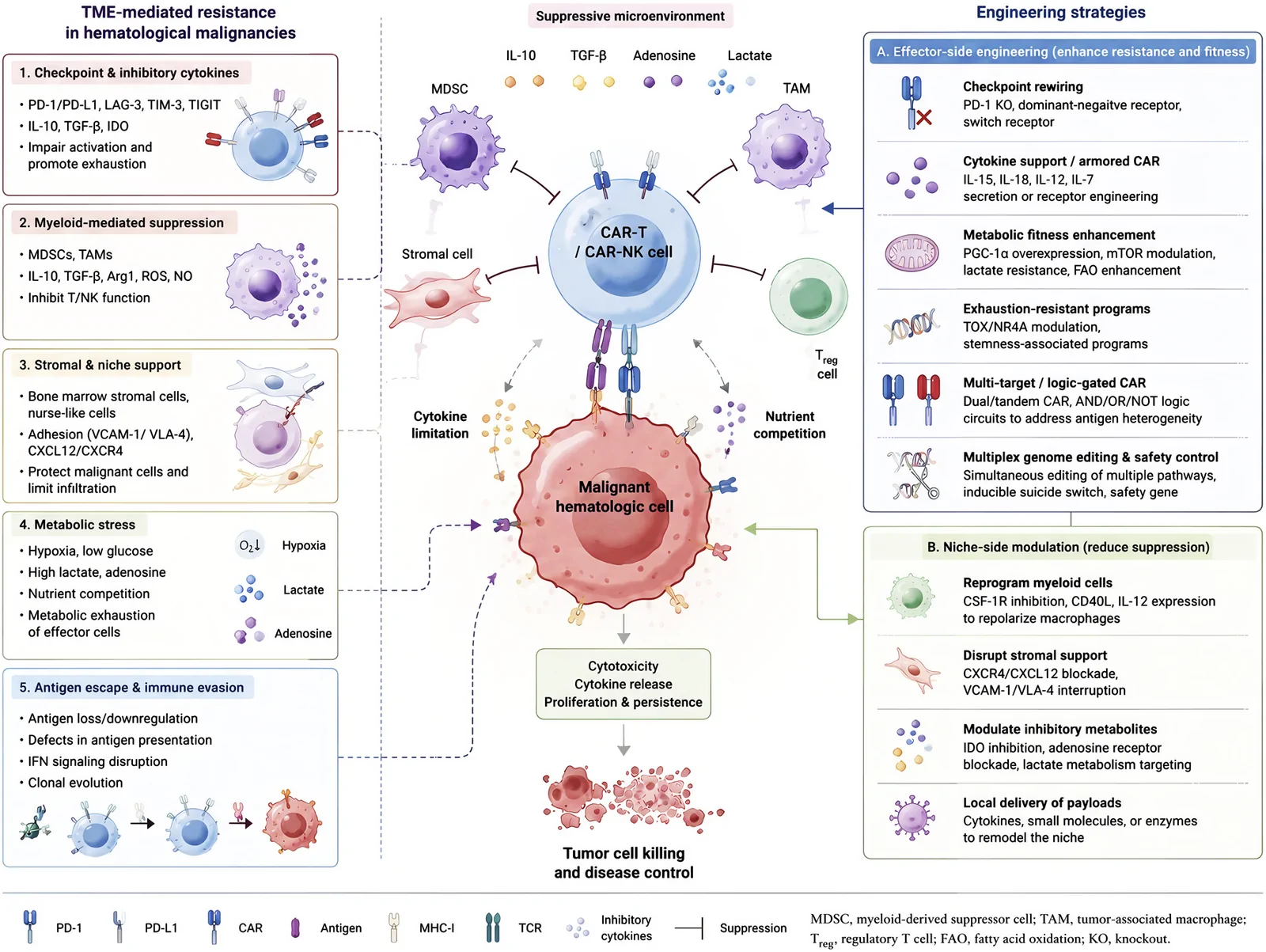
Bidirectional genome engineering strategies to overcome tumor microenvironment (TME) resistance in hematological malignancies.

Beyond tumor-intrinsic immune escape, resistance to engineered cellular therapy is also shaped by the tumor microenvironment (TME). In hematological malignancies, this suppressive environment is often organized within bone marrow, lymph node, or disease-specific immune niches, where CAR-T and CAR-NK cells are exposed to checkpoint signaling, cytokine limitation, myeloid-mediated suppression, stromal support, and metabolic stress (Islam et al., 2025). These factors can impair effector-cell expansion, accelerate exhaustion, and limit persistence even when antigen recognition and CAR signaling remain intact (Yang et al., 2024) Functional genomics and genome engineering therefore provide a rationale for a bidirectional strategy: engineered effector cells can be modified to better tolerate suppressive cues, while selected tumor or microenvironmental components may be targeted to reduce local resistance pressure (Zhang F. et al., 2025). The major microenvironmental barriers and corresponding bidirectional engineering strategies are summarized in Figure 2. This model highlights that TME resistance in hematological malignancies should be addressed through both effector-side engineering and niche-side modulation rather than by enhancing CAR signaling alone.

### 4.1 Checkpoint-dominated suppression and effector cell genome editing

Checkpoint ligand signaling, such as PD-L1–mediated inhibition, promotes chronic effector exhaustion through sustained inhibitory receptor engagement (Allela et al., 2025). Genome editing approaches—including CRISPR-mediated disruption of inhibitory receptors such as PD-1, CTLA-4, and LAG-3 — have been shown to enhance resistance of CAR-T and CAR-NK cells to suppressive signaling (Legato et al., 2025; Khawar et al., 2025). Beyond simple receptor knockout, switch receptors can convert inhibitory ligand binding into activating signals, whereas dominant-negative receptor variants may attenuate inhibitory signaling without complete gene disruption (Zhang G. et al., 2025).

Multiplex editing strategies can further combine checkpoint pathway rewiring with CAR knock-in and additional functional edits, generating effector cells with greater intrinsic resistance to inhibitory microenvironments (Wang et al., 2025). This approach may be particularly relevant in high-risk hematological malignancies with checkpoint-ligand–rich immune niches, although the optimal degree of checkpoint disruption and its long-term safety still require careful clinical evaluation (De Luca et al., 2017).

### 4.2 Cytokine limitation and synthetic cytokine circuit engineering

Insufficient cytokine support within the TME can limit the expansion, persistence, and functional recovery of engineered immune cells (Zhang et al., 2024). Functional screening studies highlight cytokine signaling integrity as a key determinant of sustained activity (Maalej et al., 2023). Engineering strategies therefore aim to generate cytokine-supported or partially cytokine-autonomous immune cells through enforced receptor expression, enhanced survival signaling, or armored CAR designs that secrete supportive cytokines (Sampaio-Ribeiro et al., 2023).

Cytokine-armored CAR constructs incorporating IL-12, IL-15, or IL-18 have shown improved persistence and functional activity in preclinical and early clinical settings (Zhang et al., 2022; Miao et al., 2024). Activation-dependent designs, such as NFAT-inducible cytokine expression systems, may help restrict cytokine release to tumor-engaged sites and reduce systemic toxicity. These self-regulated support modules are especially relevant in heavily pretreated patients, in whom endogenous immune support is often impaired (Terren et al., 2021). However, their clinical use must balance improved persistence against the risks of excessive inflammation, cytokine release, or prolonged immune activation.

### 4.3 Myeloid and macrophage-mediated suppression: reprogramming the innate compartment

Tumor-associated macrophages and myeloid-derived suppressor cells represent major drivers of TME-mediated immune inhibition through production of suppressive mediators and nutrient depletion (Bottcher et al., 2018). Functional genomic studies have identified regulators of suppressive polarization states, including transcriptional and lysosomal pathway components (Barry et al., 2018).

Genome engineering is being explored as a way to reprogram innate immune compartments toward more immune-supportive states. Targeted editing of macrophage polarization regulators may enhance antigen presentation and pro-inflammatory cytokine production (Li et al., 2018). CAR-macrophage platforms extend this concept by combining tumor-directed recognition with phagocytic and immunomodulatory functions (Klichinsky et al., 2020; Walton et al., 2025). However, because most CAR-M evidence remains preclinical and has been developed largely in solid tumor settings, its role in hematological malignancies should be considered exploratory. More immediately relevant strategies include engineering CAR-T or CAR-NK cells to secrete factors that recruit, activate, or reshape supportive innate immune subsets, thereby reducing myeloid-mediated suppression within marrow or lymphoma-associated niches (Sloas et al., 2021).

### 4.4 Stromal and niche-derived barriers: targeting local supportive resistance

In hematological malignancies, stromal resistance is less often a purely physical barrier and more commonly reflects a supportive disease niche. Bone marrow stromal cells, lymph node stromal elements, vascular niches, and extracellular matrix components can provide survival signals, alter cytokine gradients, and limit immune-cell access or function (Lu et al., 2022). Perturbation studies have identified regulators of stromal signaling and matrix remodeling that may contribute to local therapeutic resistance (Zhang et al., 2022).

Engineering strategies that target stromal or niche-derived resistance aim to weaken tumor-supportive signals and improve effector-cell access to protected disease compartments (Biederstadt and Rezvani, 2021). Engineered immune cells may also be designed to deliver locally acting payloads that modify stromal signaling or improve immune-cell recruitment (Hu et al., 2018). Compared with solid tumors, where dense extracellular matrix may physically restrict infiltration, hematological malignancies more often require strategies that disrupt protective cellular crosstalk within the marrow or lymphoid microenvironment (Daher et al., 2021). This distinction is important when translating stromal-targeting concepts into CAR-T or CAR-NK therapy for blood cancers.

### 4.5 Metabolic suppression and engineering metabolic fitness

Metabolic stress represents another important constraint on engineered immune cells. Nutrient competition, hypoxia, lactate accumulation, and other suppressive metabolites can reduce effector-cell proliferation, cytokine production, and cytotoxic activity (Gang et al., 2020). Functional screens have identified metabolic checkpoints that regulate immune-cell fitness under nutrient-limited or inflammatory conditions (Xie et al., 2020).

Metabolic engineering strategies include enhancing mitochondrial resilience, enabling alternative nutrient utilization pathways, and strengthening oxidative phosphorylation programs (Gong et al., 2021). Editing transporters and metabolic enzymes in engineered immune cells has been shown in preclinical models to improve functional persistence under nutrient-limited conditions (Klichinsky et al., 2020; Rezvani et al., 2017). Complementary approaches target tumor or stromal metabolic enzymes to reduce production of suppressive metabolites. Metabolic rewiring should therefore be viewed as a supportive axis alongside receptor, checkpoint, and cytokine engineering, particularly for CAR-NK platforms, which may have greater intrinsic metabolic flexibility.

### 4.6 Bidirectional engineering as a unifying framework

Taken together, TME-mediated resistance in hematological malignancies is unlikely to be overcome by improving CAR signaling alone. Effector-cell edits, including checkpoint rewiring, cytokine support, and metabolic optimization, can increase functional autonomy. At the same time, selected tumor- or niche-directed interventions may reduce suppressive pressure in marrow, lymph node, or other disease-specific microenvironments (Zhang et al., 2024). Multiplex editing technologies and functional resistance maps make it possible to coordinate these interventions across several levels of resistance biology. This bidirectional approach is most relevant in high-risk or relapse-prone hematological malignancies, where suppressive immune niches and adaptive tumor evolution frequently coexist (Sampaio-Ribeiro et al., 2023). Rather than serving as a universal solution, bidirectional engineering should be viewed as a strategy for matching the dominant microenvironmental barrier with an appropriate effector-cell or niche-directed modification.

## 5 CAR-NK and universal off-the-shelf platforms in escape-prone and high-risk settings

Autologous CAR-T cell therapy has achieved major clinical advances in hematological malignancies, but its limitations become more apparent in escape-prone and TME-suppressed disease settings. Manufacturing delays, variable product quality in heavily pretreated patients, prior therapy–associated T-cell dysfunction, and limited feasibility of repeated dosing may all compromise treatment delivery or durability (Jain et al., 2024; Neelapu, 2023). These challenges have supported the development of alternative engineered immune platforms, particularly CAR-NK cells and universal off-the-shelf gene-edited products (Kong et al., 2025).

Genome engineering is central to these approaches because it can reduce immunologic incompatibility, improve product consistency, and introduce resistance-countering modules (Wang et al., 2025). These platforms should therefore be viewed not only as logistical alternatives to autologous CAR-T therapy, but also as potential biologic strategies for selected high-risk or relapse-prone disease contexts (Zhao et al., 2026).

### 5.1 CAR-NK cells: innate cytotoxicity and reduced dependence on single-antigen recognition

Natural killer (NK) cells provide a layered recognition system that combines CAR-directed targeting with endogenous activating and inhibitory receptors capable of sensing cellular stress and missing-self signals (Legato et al., 2025). This biology may reduce exclusive dependence on a single tumor antigen and could partially limit selective pressure for antigen-negative relapse (Khawar et al., 2025).

From a resistance-oriented perspective, CAR-NK platforms have several theoretical and early clinical advantages, including CAR-independent cytotoxicity, low risk of graft-versus-host disease, generally lower rates of severe cytokine release syndrome and neurotoxicity, compatibility with allogeneic sourcing, and the possibility of repeated dosing (De Luca et al., 2017; Zhao et al., 2025). These features are particularly relevant for heavily pretreated patients or those with poor autologous T-cell fitness (Zhang et al., 2022).

Genome engineering may further improve CAR-NK performance through cytokine-support circuits, signaling rewiring, and metabolic optimization designed to enhance persistence and functional resilience. Early-phase clinical studies of CAR-NK therapies targeting CD33, CD123, NKG2D ligands, and other AML-associated antigens have demonstrated encouraging safety profiles and preliminary antileukemic activity. Emerging allogeneic CAR-NK products, including NKX101, have shown early clinical responses in relapsed or refractory AML and higher-risk MDS, supporting the feasibility of off-the-shelf NK-cell immunotherapy in myeloid malignancies. Furthermore, induced pluripotent stem cell (iPSC)-derived CAR-NK platforms such as FT596 provide scalable manufacturing, standardized genome engineering, and the potential for repeated dosing, highlighting a promising strategy for overcoming limitations associated with autologous cellular therapies (Yang et al., 2024; Ghobadi et al., 2025a; Huang et al., 2025; Bahramloo et al., 2024).

Despite these advantages, CAR-NK therapy has important limitations. Compared with conventional autologous CAR-T cells, CAR-NK cells generally show shorter *in vivo* persistence and more limited antigen-driven expansion, which may reduce durability unless cytokine support, repeat dosing, or persistence-enhancing engineering is incorporated. NK cells can also be more difficult to genetically modify than T cells, and manufacturing may be affected by source-dependent variability, transduction or editing efficiency, cryopreservation-associated functional loss, and challenges in obtaining sufficient cell numbers. Trafficking to protected marrow, lymph node, extramedullary or central nervous system compartments may be inadequate in some disease contexts. In addition, excessive exogenous cytokine support may increase toxicity or alter NK-cell differentiation, whereas insufficient support may compromise activity. These limitations mean that CAR-NK cells should be positioned as a complementary platform with distinct biological strengths rather than as a universally superior substitute for CAR-T therapy.

However, the durability of CAR-NK responses, optimal cytokine support strategy, and persistence-enhancing design remain less established than those of approved autologous CAR-T products. Current data therefore support CAR-NK cells as a promising platform for relapse-prone disease, but further prospective studies are needed to define where they should be positioned relative to CAR-T therapy, bispecific antibodies, and transplantation-based strategies.

### 5.2 Universal gene-edited CAR-T products: overcoming autologous manufacturing and fitness limitations

Universal allogeneic CAR-T platforms aim to decouple therapy from patient-specific manufacturing by using donor-derived T cells that are genome-edited to reduce graft-versus-host reactivity and host rejection (Garcia-Robledo et al., 2025; Jiang et al., 2025). Common approaches include disruption of endogenous T-cell receptor loci and modification of histocompatibility pathways through multiplex CRISPR editing or base editing, enabling more standardized off-the-shelf production (Murray et al., 2025).

From a resistance-matching perspective, these platforms may offer two practical advantages. First, donor-derived cells may provide a more controlled baseline immune fitness than autologous T cells collected from heavily pretreated patients (Cheema et al., 2026). Second, large-scale manufacturing allows multiplex resistance-countering edits, such as checkpoint rewiring, exhaustion resistance, or metabolic enhancement, to be incorporated without extending individualized production timelines (Wang et al., 2025).

Early clinical reports of gene-edited allogeneic CAR-T products in T-cell and plasma cell malignancies have shown encouraging composite remission rates in heavily pretreated populations, often with high MRD-negative fractions and manageable toxicity profiles (Ghobadi et al., 2025b). Base-edited lineage-directed products have also induced remissions sufficient to bridge selected patients to allogeneic transplantation (Chiesa et al., 2026). BCMA-directed allogeneic CAR-T platforms have reported high objective response rates in interim analyses under optimized lymphodepletion regimens, with relatively low observed rejection signals (Tseng et al., 2025). Multiplex CRISPR-edited products targeting T-lineage antigens have similarly shown promising response rates and low rates of graft-versus-host disease in early-phase updates (Alexander et al., 2025). These findings support the clinical feasibility of universal CAR-T systems in high-risk disease settings (Silbert and Lamble, 2025). Nevertheless, their long-term persistence, host-versus-graft rejection, lymphodepletion requirements, genomic safety, and comparative efficacy against established autologous CAR-T products remain key unresolved questions.

### 5.3 iPSC-derived and renewable engineered immune cell sources

Induced pluripotent stem cell (iPSC)–derived immune platforms provide a renewable and highly programmable source for engineered cellular therapies (Ghobadi et al., 2025a; Wei et al., 2025). Genome edits can be introduced at the pluripotent stage, clonally validated, and propagated through controlled differentiation into NK-like or T-like effector populations. This strategy may improve product uniformity and facilitate the incorporation of multiple edits or synthetic circuits before clinical-scale manufacturing.

iPSC-derived platforms offer several potential advantages, including clonal genetic consistency, renewable supply, compatibility with repeated dosing, and standardized product design. Recent trial and conference updates of iPSC-derived CAR-NK products have reported encouraging persistence, multifunctional engineering capacity, and scalable manufacturing performance in hematologic malignancies (Wei et al., 2025). Preclinical dual-target and multi-module iPSC-derived constructs further suggest that this platform can accommodate more complex resistance-countering designs (Hou et al., 2025). However, iPSC-derived products still face important translational challenges, including differentiation fidelity, functional maturity, long-term *in vivo* durability, tumorigenic safety, and batch-to-batch consistency. Therefore, iPSC-based platforms should be considered an important off-the-shelf engineering backbone, but their optimal clinical role in relapse-prone hematological malignancies remains to be defined.

### 5.4 Platform selection as a function of resistance biology

Platform selection should increasingly be guided by resistance biology rather than by availability alone (Garcia-Robledo et al., 2025). Different resistance patterns may favor different cellular backbones. Antigen-heterogeneous disease may benefit from multi-target designs or NK-based systems with innate recognition redundancy (Zhao et al., 2026). Settings dominated by poor autologous T-cell fitness may favor donor-derived or stem-cell-derived products (Legato et al., 2025). Clinical scenarios requiring repeated dosing may be better suited to CAR-NK or other off-the-shelf approaches, particularly when inflammatory toxicity needs to be minimized.

Genome engineering enables these choices by reducing compatibility barriers and allowing resistance-countering edits to be embedded into different platforms (Wang et al., 2025). In this sense, platform selection becomes part of precision immunotherapy design, together with antigen choice, CAR architecture, and editing strategy (Khawar et al., 2025). At the same time, resistance biology should not be used as a purely theoretical classification. For platform selection to become clinically useful, resistance features such as antigen heterogeneity, T-cell exhaustion, checkpoint-ligand dominance, or marrow niche suppression will need to be measured reproducibly before treatment and linked to prospective outcome data.

### 5.5 Convergence toward modular but clinically grounded engineering principles

Although CAR-T, CAR-NK, universal allogeneic, and stem-cell-derived platforms differ biologically, several engineering principles are now shared across these systems (Mohammad et al., 2024). These include multiplex editing, logic-gated recognition, checkpoint pathway rewiring, cytokine-support circuits, and metabolic fitness enhancement. Genome engineering provides a common technical layer that allows selected resistance-countering modules to be adapted across different cellular backbones (Bayat et al., 2026; Hosking et al., 2025).

This convergence supports a more modular approach to engineered cellular therapy, in which platform backbone, edit modules, and circuit logic can be selected according to disease biology and clinical contextt (Xu et al., 2026). However, the idea of “plug-and-play” cellular therapy should be interpreted cautiously. Greater engineering complexity may improve resistance coverage, but it can also increase manufacturing burden, regulatory complexity, genomic safety concerns, and functional unpredictability. The next step is therefore not simply to add more modules, but to identify which edits are biologically necessary, clinically measurable, and safe enough for a given high-risk disease setting. The major biological rationales, best-fit resistance settings, advantages, limitations, and translational status of these platforms are summarized in Table 1.

**TABLE 1:** Platform selection and translational considerations for genome-engineered cellular therapies.

Platform	Biological rationale	Best-fit setting	Advantages	Limitations	Status
Autologous CAR-T	Patient-derived T cells with validated clinical efficacy	CD19^+^ lymphoma/B-ALL; BCMA + MM with adequate T-cell fitness	Established efficacy, durable remissions in selected patients	Manufacturing delay, poor product quality after heavy pretreatment, limited repeat dosing	Clinically validated
Genome-edited autologous CAR-T	Edited T cells with enhanced persistence or suppression resistance	Checkpoint-rich or exhaustion-prone relapse	Individualized product with resistance-countering edits	Editing complexity, cost, manufacturing variability	Early clinical/translational
Universal allogeneic CAR-T	Donor-derived edited T cells	Poor autologous T-cell fitness; urgent treatment need	Off-the-shelf access, standardized product, multiplex edit capacity	Rejection, GVHD risk, lymphodepletion dependence, persistence uncertainty	Early clinical
CAR-NK	Innate recognition plus CAR targeting	Antigen-heterogeneous disease; repeat dosing; toxicity-sensitive patients	Lower GVHD risk, lower severe CRS/ICANS signal, allogeneic compatibility	Limited persistence, cytokine support required, durability unclear	Early clinical
iPSC-derived CAR-NK/CAR-T-like cells	Renewable clonally edited source	Standardized repeat dosing; multi-edit platform design	Scalable, uniform, prevalidated edits	Differentiation fidelity, maturity, tumorigenic safety, long-term durability	Early clinical/preclinical
Multiplex/circuit-engineered products	Multiple resistance layers addressed in one product	Polygenic escape, antigen heterogeneity plus TME suppression	Broader resistance coverage	Genomic safety, functional unpredictability, complex QC	Mostly translational/early clinical

## 6 Implications for high-risk disease and precision immunotherapy framework

Resistance architecture refers to the integrated set of tumor-intrinsic, microenvironmental, and host-related mechanisms that collectively determine resistance to engineered cellular therapies within a given disease context. Rather than representing a single resistance pathway, resistance architecture encompasses multiple interacting resistance modules, including antigen escape, checkpoint-mediated suppression, impaired inflammatory signaling, metabolic stress, stromal protection, and effector-cell dysfunction.

Resistance-matched design refers to the rational selection of CAR architectures, genome-engineering modules, and cellular platforms according to the dominant resistance architecture identified through functional genomics, molecular profiling, immune phenotyping, and clinical assessment. In this framework, engineered cellular therapies are not optimized solely for maximal potency, but are instead tailored to the principal biological mechanisms that limit therapeutic efficacy in individual disease settings.

High-risk hematologic malignancies remain a major unmet need in cellular immunotherapy. Although engineered immune-cell therapies have produced important responses in selected patients, outcomes remain inconsistent in genetically unstable, heavily pretreated, and immune-evasive disease settings. Relapse remains common in aggressive subtypes such as DLBCL and AML, even after apparently effective cellular therapy (Aymard et al., 2026). These observations suggest that high-risk disease should not be defined only by tumor burden, cytogenetic abnormalities, or prior lines of therapy, but also by the dominant resistance mechanisms that may limit engineered-cell efficacy.

### 6.1 Defining resistance architecture in high-risk hematologic malignancies

Traditional high-risk classifications based on clinical stage, cytogenetics, molecular abnormalities, and treatment history remain essential. However, these variables do not always explain why some patients relapse rapidly after CAR-T or CAR-NK therapy, whereas others achieve durable responses. Genome-scale functional studies suggest that treatment failure is often shaped by immune resistance modules that cut across classical disease categories (Aymard et al., 2026; Zugasti et al., 2025). These modules include antigen heterogeneity, impaired interferon and antigen-presentation pathways, checkpoint-ligand dominance, suppressive myeloid or stromal niches, prior therapy–associated immune dysfunction, and activation of polygenic survival programs.

Functional genome engineering screens and perturbation maps make it possible to define these resistance modules experimentally rather than relying only on descriptive biomarkers (Chen et al., 2025). Single-cell and functional studies increasingly suggest that post–cell therapy relapse is often associated with combined resistance features rather than a single driver mutation. Under this view, high-risk disease can be reframed as escape-prone, suppression-dominant, or effector-limited, depending on the dominant barrier to cellular therapy activity. This resistance-architecture model is particularly useful in relapsed and refractory settings, where prior therapies impose selective pressure, enrich immune-resistant clones, and impair host immune fitness. It does not replace conventional clinical and molecular risk stratification. Rather, it adds a functional layer that may better explain why similar patients respond differently to the same engineered cellular product.

### 6.2 Mechanism-matched selection of engineered cell therapy designs

A key implication of resistance mapping is that engineered cell therapy design should not be uniform across all high-risk patients. Different resistance drivers suggest different engineering priorities, drawing on the strategy classes described in Sections 3–5 (Putigna et al., 2025).

In practical terms, antigen heterogeneity may favor multi-target or logic-gated CAR designs; antigen instability may support platforms with recognition redundancy, such as NK-based systems; checkpoint-dominant suppression may favor checkpoint-rewired or knockout effector cells; cytokine-poor niches may require cytokine-armored constructs; metabolically hostile niches may benefit from metabolically reinforced effector cells; poor autologous T-cell quality may favor donor-derived or renewable platforms; and polygenic escape risk may justify multiplex or circuit-level engineering.

In this framework, genome engineering acts as a translation layer between resistance biology and product design. The central question is not which CAR configuration is universally optimal, but which engineered configuration best matches the dominant resistance drivers in a given disease context. For example, in high-risk leukemias with antigen heterogeneity and immune suppression, a single-target autologous CAR-T product may be biologically insufficient, whereas multiplex-armored or NK-based approaches may offer theoretical advantages.

Operationally, resistance-matched design requires integration of multiple data layers. Functional genomic screens, genomic sequencing, transcriptomics, immune profiling, and clinical characteristics collectively contribute to the identification of dominant resistance architectures. These resistance features can then be categorized into major classes, including antigen escape, checkpoint-mediated suppression, cytokine limitation, stromal resistance, metabolic stress, and host-cell dysfunction. Based on the dominant resistance module and its associated biomarkers, corresponding engineering strategies and cellular platforms may be selected. For example, reduced antigen density may support multi-target CAR designs, elevated checkpoint expression may favor checkpoint-rewired effector cells, whereas impaired autologous immune fitness may support consideration of allogeneic or renewable cellular platforms.

**FIGURE 3:**
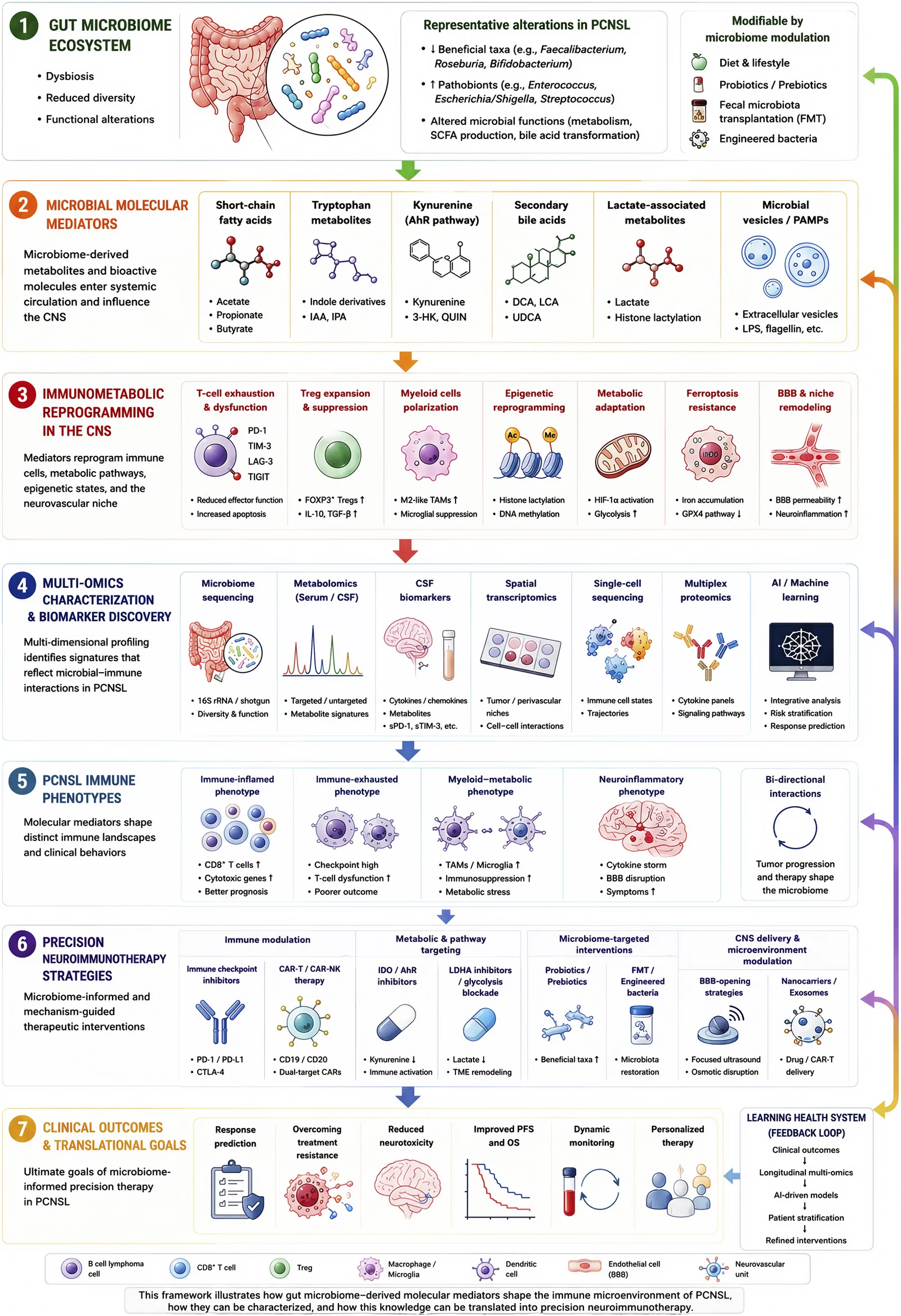
Mechanistic-translational framework for microbiome-informed precision immunotherapy in primary central nervous system lymphoma (PCNSL).

For practical implementation, this mechanism-to-design logic is organized into a resistance-matched engineering matrix (Table 2), which is graphically illustrated in Figure 3 (Chen et al., 2025). The matrix links major resistance modules with candidate engineering strategies, potential biomarkers, platform options, and key translational considerations, thereby providing a structured workflow for translating resistance profiling into engineering decisions. Rather than serving as a fixed treatment algorithm, the framework is intended as a conceptual decision-support model that connects resistance biology with engineering strategy selection and may help guide future experimental and clinical development. In its current form, this framework is primarily intended for translational research and biomarker-driven clinical studies. Routine implementation will depend on the availability of standardized molecular profiling, functional annotation platforms, and prospective validation in disease-specific patient cohorts.

**TABLE 2:** Resistance-matched engineering strategies for CAR-T and CAR-NK therapies in hematological malignancies.

Resistance module	Disease context	Biomarkers	Engineering strategy	Platform	Caveat
Antigen loss/antigen-low relapse	CD19 CAR-T relapse in B-cell malignancies; BCMA relapse in MM	Flow cytometry, IHC, single-cell antigen profiling	Dual-target CAR, tandem CAR, logic-gated CAR	CAR-T/CAR-NK	Antigen loss may coexist with poor effector fitness
Antigen-presentation/IFN pathway defects	Immune-evasive lymphoma/leukemia	IFN signature, JAK–STAT alteration, MHC-related gene expression	MHC-independent targeting, surfaceome-guided antigen selection	CAR-T/CAR-NK	CAR recognition bypasses MHC but does not restore immune inflammation
Checkpoint-dominant suppression	PD-L1-rich lymphoma niche	PD-L1, PD-1, LAG-3, exhausted T-cell profile	PD-1 knockout, switch receptor, dominant-negative receptor	CAR-T/CAR-NK	Excessive checkpoint disruption may affect safety
Cytokine-poor niche	Heavily pretreated patients, marrow-suppressed disease	Low cytokine support, poor expansion kinetics	IL-15/IL-18 armored CAR, cytokine receptor rewiring	CAR-T/CAR-NK	Inflammatory toxicity must be monitored
Myeloid/macrophage suppression	Lymphoma-associated macrophage-rich niche; AML marrow niche	TAM/MDSC markers, IL-10, TGF-β, arginine metabolism	Cytokine secretion, innate immune recruitment, niche modulation	CAR-T/CAR-NK	CAR-M remains exploratory in hematologic malignancies
Metabolic stress	Marrow niche, hypoxic or lactate-rich disease	Hypoxia signature, lactate, mitochondrial fitness	Metabolic rewiring, mitochondrial support	CAR-NK/CAR-T	Mostly preclinical
Poor autologous T-cell fitness	Heavily pretreated relapse	T-cell exhaustion phenotype, low T-cell yield	Allogeneic CAR-T, CAR-NK, iPSC-derived products	Universal/CAR-NK/iPSC	Persistence and rejection remain unresolved
Polygenic escape	High-risk AML, aggressive lymphoma	Multi-omics, CRISPR maps, clonal evolution	Multiplex editing, circuit-level engineering	Selected platforms	Complexity increases manufacturing and safety burden

### 6.3 Case illustration: resistance-matched design in relapsed DLBCL

To illustrate how the proposed framework may be operationalized, consider a patient with relapsed diffuse large B-cell lymphoma (DLBCL) following multiple prior lines of therapy. Molecular and immune profiling demonstrates reduced CD19 expression, elevated PD-L1 levels, exhaustion-associated transcriptional signatures, and impaired autologous T-cell fitness (Majzner and Mackall, 2018; Huang et al., 2026).

These findings collectively define a resistance architecture characterized by antigen escape, checkpoint-mediated suppression, and host-cell dysfunction. Based on the resistance-matched framework, several engineering priorities emerge. Antigen instability may support the use of dual-target CAR constructs (e.g., CD19/CD20) to reduce the risk of antigen-negative relapse (Tong et al., 2020; Han et al., 2019). Elevated checkpoint signaling may favor PD-1-disrupted or checkpoint-rewired effector cells (Rupp et al., 2017; Cherkassky et al., 2016). Impaired autologous T-cell fitness may further support consideration of allogeneic CAR-NK or renewable cellular platforms (Yang et al., 2024; Ghobadi et al., 2025a).

This example illustrates how functional genomics, molecular profiling, and immune phenotyping can be integrated to guide engineering decisions and platform selection. Although prospective validation remains necessary, such an approach highlights the potential of resistance-matched design as a mechanism-informed strategy for precision cellular immunotherapy. Importantly, this example is intended to illustrate the conceptual workflow of resistance-matched design rather than to recommend a specific clinical treatment strategy.

### 6.4 Integrating multi-omics and functional genomics into therapy selection

Precision engineered immunotherapy requires integration of several data layers. Genomic sequencing, transcriptomics, epigenomic profiling, and immune landscape analysis can describe resistance-associated features, whereas CRISPR functional screening and perturbation platforms can help identify which features are causally involved in treatment failure (Gong et al., 2025; Yang et al., 2023). Together, these approaches may support a staged selection framework.

A practical workflow may include baseline multi-omics and immune profiling; assessment of antigen expression, signaling defects, and TME suppressive features; functional annotation using perturbation or CRISPR reference maps; selection of CAR architecture and editing modules; platform choice among autologous, universal, NK-based, or stem-cell–derived systems; and serial monitoring for emerging escape patterns after treatment.

Patient-derived organoids, xenografts, and edited co-culture systems may provide additional *ex vivo* testing platforms for selected high-risk cases. (Logun et al., 2025). These models can help evaluate whether a proposed resistance-countering strategy is biologically plausible before clinical deployment. However, their routine clinical use remains limited by turnaround time, standardization, cost, and the need for prospective validation. Therefore, multi-omics and functional testing should be viewed as tools to refine treatment selection, not as immediate replacements for established clinical decision-making.

### 6.5 *In Vivo* genome editing and combination precision strategies

An emerging extension of resistance-guided therapy involves combining engineered immune cells with *in vivo* genome editing approaches that target tumor or microenvironmental resistance nodes (Meng et al., 2025; Lemgart et al., 2026). Non-viral delivery systems, including lipid nanoparticles (LNPs), polymeric nanoparticles, and inorganic nanocarriers, have made localized editing of selected pathways increasingly feasible in preclinical and early translational settings (Ang et al., 2026).

Conceptually, this strategy creates a dual-layer intervention: engineered immune cells provide targeted cytotoxicity, while localized genome editing reduces escape signaling or suppressive cues within the tumor niche. Such approaches may be relevant for anatomically or microenvironmentally protected disease compartments, including bone marrow–dominant malignancies.

At present, however, *in vivo* editing in this context remains largely experimental. Key barriers include delivery specificity, off-target effects, editing efficiency, immunogenicity, and the difficulty of controlling edited cells or stromal targets after administration. Therefore, combination strategies involving *in vivo* editing should be presented as a future direction rather than an established component of cellular immunotherapy (Launspach et al., 2025).

### 6.6 A prototype precision engineered immunotherapy algorithm

Taken together, current evidence supports a prototype framework for resistance-guided engineered cell therapy in high-risk hematologic malignancies. The main steps include defining resistance features through multi-omics and functional annotation, identifying dominant immune escape and TME modules, selecting CAR architecture and editing strategies that match these modules, choosing a cellular platform according to immune fitness and dosing needs, incorporating multiplex edits only when resistance is clearly multilayered, and monitoring for evolutionary escape after treatment (Gong et al., 2025; Wen et al., 2026).

As summarized in Figure 3 and further organized in Table 2, this approach reframes genome-engineered cell therapy from a fixed product model into a more adaptable therapeutic strategy guided by resistance biology and functional genomics (Zugasti et al., 2025). Its immediate value is not to provide a ready-made clinical algorithm, but to clarify how future trials could stratify patients, select engineering strategies, and test whether resistance-matched design improves durability in high-risk disease.

## 7 Translational, safety, and regulatory challenges in genome-engineered cell therapy

As genome-engineered cellular therapies become more complex, their clinical translation increasingly depends on whether safety, product consistency, and long-term behavior can be controlled reproducibly. The challenge is no longer limited to generating edited CAR-T or CAR-NK cells with improved function; it is to demonstrate that multiplex edits, synthetic circuits, and potential *in vivo* editing strategies do not introduce unacceptable genomic, functional, or manufacturing uncertainty. This is particularly important for universal, stem-cell–derived, and heavily multiplexed products, where multiple engineering layers may interact in ways that are difficult to predict from single-edit models (Wang et al., 2025). Therefore, translational development requires coordinated assessment of genomic integrity, delivery specificity, post-infusion stability, manufacturing reproducibility, and risk-adaptive clinical evaluation.

### 7.1 Genomic integrity and off-target risk in multiplex editing

Multiplex genome editing, such as combined checkpoint disruption, cytokine circuit insertion, and CAR knock-in, introduces cumulative genomic risk beyond conventional single-locus modification (Wang et al., 2025). Reported concerns include off-target cleavage, large deletions, chromosomal rearrangements, translocations, and non-linear fitness effects caused by interactions among edits (Lei et al., 2024; Murray et al., 2025). Here, non-linear fitness effects refer to situations in which the combined biological consequences of multiple genetic modifications differ from the simple sum of their individual effects, resulting in synergistic or antagonistic influences on cellular fitness, persistence, or functionality. Although high-fidelity editors, optimized guide RNAs, and improved editing protocols can reduce off-target activity, multiplex configurations remain more difficult to characterize than single-edit products. Structural variants may still occur in a minority of edited cells and may not be fully captured by standard targeted assays (Administration, 2024).

Mitigation strategies include computational and AI-assisted guide design, transient editor delivery, orthogonal validation assays, and comprehensive genomic quality control using unbiased off-target mapping and long-read sequencing. Increasingly, clinical protocols incorporate longitudinal genomic monitoring before and after infusion, particularly for multiplex-edited universal and stem-cell–derived platforms. Recent regulatory guidance also emphasizes edit-resolved genomic characterization for highly engineered products.

### 7.2 Delivery control: *Ex Vivo* versus in vivo editing

*Ex vivo* editing offers relatively controlled exposure to editing reagents and allows extensive product testing before infusion. This controllability has supported most clinical progress in genome-edited cellular therapy to date (Gao et al., 2026). In contrast, *in vivo* genome editing faces additional uncertainty related to biodistribution, target-cell specificity, delivery-vector immunogenicity, editing efficiency, and persistence of the editing machinery (Ang et al., 2026). Viral and non-viral delivery systems each involve tradeoffs in efficiency, tissue tropism, payload capacity, and safety.

For hematological malignancies, *in vivo* editing should be viewed as a future extension rather than a near-term replacement for *ex vivo* manufacturing. Although nanoparticle and vector-based systems are improving, selective delivery to the intended tumor, immune, or microenvironmental compartment remains difficult (Song et al., 2025). This limitation is especially relevant when proposed editing targets are located within stromal or immune-suppressive niches rather than in the infused cellular product itself. Delivery feasibility should therefore be considered early in engineering design, because it determines which resistance pathways are realistically actionable *in vivo*.

### 7.3 Functional stability and post-infusion evolution

Engineered immune cells remain biologically dynamic after infusion. Antigen exposure, inflammatory cues, homeostatic cytokines, and suppressive microenvironmental signals can reshape their differentiation state, exhaustion trajectory, metabolic profile, and persistence (Wu et al., 2026). A product that appears potent at release may not retain the same functional phenotype after prolonged antigen stimulation or repeated exposure to suppressive niches. This issue is particularly relevant for edits that alter checkpoint signaling, cytokine responsiveness, or survival pathways. Such modifications may improve short-term activity but could also change exhaustion kinetics, expansion behavior, or long-term clonal fitness in ways that vary across patients. Therefore, durability assessment should include extended stress testing, serial functional assays, phenotypic tracking, and *in vivo* models that reflect repeated antigen stimulation. For renewable or stem-cell–derived platforms, additional validation of lineage fidelity, differentiation stability, and long-term state maintenance is required. Single-cell profiling and longitudinal phenotypic mapping may help identify phenotype drift and functional instability before or after clinical administration (Fang et al., 2025).

### 7.4 Manufacturing consistency and release criteria

As engineered cell products incorporate more edits and circuit modules, manufacturing consistency becomes a central translational challenge (Marta and Costa, 2025). Variability may arise in editing efficiency, edit combinations, expansion behavior, and phenotypic composition. Advanced quality control approaches now include edit-resolved next-generation sequencing, circuit-specific molecular assays, high-dimensional phenotyping, and automated process monitoring (Cappabianca et al., 2024).

Regulatory expectations are evolving toward more granular, edit-resolved release criteria for complex engineered products, particularly universal and multiplex-edited platforms.

### 7.5 Ethical and regulatory considerations

Somatic genome editing approaches reduce the risk of germline transmission, but long-term safety monitoring remains essential (Administration, 2024). Ethical and regulatory considerations include oncogenic risk surveillance, duration of follow-up, cumulative risk from multiplex editing, and equitable access to advanced therapies (Ortiz-Bueno et al., 2026). Emerging regulatory frameworks emphasize long-term patient monitoring, genomic surveillance, adaptive trial designs, and platform-level evaluation rather than single-product assessment alone.

### 7.6 Risk-adaptive development and clinical trial design

Engineering complexity should be matched to disease severity, unmet need, and the maturity of supporting evidence. More complex multiplex or circuit-level products may be justified in refractory, high-risk disease settings, where conventional options are limited and resistance mechanisms are clearly multilayered. In earlier-line settings, however, the same level of engineering complexity may not be appropriate unless the added edits provide a measurable clinical advantage. This risk-adaptive principle aligns with the resistance-matched framework discussed in Section 6.

Clinical trial design will need to evolve accordingly. Adaptive protocols, biomarker-guided stratification, modular dose escalation, and resistance-stratified trial structures may help determine which engineered modules are necessary for specific patient populations (Muller et al., 2026). Rather than testing only whether a product is active, next-generation trials should also test whether the proposed resistance-matched design improves durability, safety, or response consistency compared with simpler cellular platforms. Basket-style or platform-based studies may be useful when the same resistance mechanism appears across several hematological malignancies, but they should be supported by clear biological entry criteria and standardized correlative studies.

Overall, translational success will depend on balancing innovation with controllability. The goal is not to maximize engineering complexity, but to identify the minimum set of edits or circuits that meaningfully improves resistance coverage while remaining manufacturable, monitorable, and clinically safe.

## 8 Future directions: programmable and adaptive precision cell therapy

The future development of genome-engineered cellular therapy will depend less on the number of edits introduced into a product than on whether each modification addresses a measurable resistance mechanism. Genome engineering, functional genomics, synthetic biology, systems immunology, and artificial intelligence are increasingly being integrated to guide CAR-T, CAR-NK, universal allogeneic, and stem-cell–derived platforms (Wen et al., 2026). However, the clinical value of these approaches will depend on whether programmable designs can be manufactured reproducibly, monitored longitudinally, and validated in defined high-risk hematological malignancy populations.

Programmable genetic circuits remain an important direction. Logic-gated receptors incorporating AND, OR, or NOT functions, inducible activation modules, and feedback-controlled cytokine release systems may improve tumor selectivity in antigen-heterogeneous disease settings (Lei et al., 2025). Genome editing enables more stable integration of such circuits across different cellular platforms. Nevertheless, circuit-based products will be clinically useful only if their activation thresholds, persistence, and safety-control mechanisms are predictable across patients. At present, many of these systems remain in preclinical or early clinical development, and their superiority over simpler multi-target designs has not yet been established.

Precision editing technologies may further refine engineered cell design. Base editing, prime editing, and epigenome editing allow more tunable genetic modulation than conventional nuclease-based disruption (Lei et al., 2024). These approaches may be particularly useful for immune regulatory pathways, such as checkpoint or interferon-related signaling, where partial modulation may be preferable to complete gene knockout. Even so, improved editing precision does not eliminate the need for functional validation. The key question is not only whether an edit can be made more accurately, but whether the edited pathway produces a durable and clinically meaningful improvement in cellular therapy performance.

Artificial intelligence–assisted design is likely to play an increasing role in target prioritization, resistance prediction, edit-combination selection, and receptor optimization. Machine learning models trained on CRISPR screening datasets, multi-omics profiles, and clinical outcome data may help reduce empirical trial-and-error in engineered cell development (Ansari et al., 2026). However, AI-assisted design should be viewed as a prioritization tool rather than a substitute for experimental and clinical validation. Current models remain limited by heterogeneous datasets, small clinical cohorts, variable manufacturing conditions, and insufficient prospective testing. For this reason, AI-generated targets or edit combinations should be confirmed through functional screening, disease-specific models, and clinical correlative studies before being incorporated into therapeutic products.

Adaptive monitoring will also become increasingly important. Longitudinal biomarkers such as circulating tumor DNA, antigen-expression dynamics, immune repertoire changes, and single-cell state profiling may help detect emerging escape patterns after cellular therapy. These data could guide sequential treatment decisions, combination strategies, or selection of a second engineered platform. However, a truly closed-loop therapeutic model remains premature. Before adaptive cellular therapy can be implemented clinically, biomarker thresholds, sampling intervals, response-adaptation rules, and intervention timing will need to be standardized and tested prospectively.

Platform convergence is another important trend, but it should not be interpreted as platform equivalence. CAR-T, CAR-NK, universal allogeneic, and iPSC-derived products increasingly share modular engineering components, including multiplex edit cassettes, logic circuits, cytokine-support modules, and metabolic fitness programs. (Joechner et al., 2025). At the same time, these platforms differ substantially in persistence, cytotoxic mechanisms, inflammatory toxicity, manufacturing constraints, and feasibility of repeat dosing. Future design should therefore match engineering modules to both resistance biology and platform-specific strengths, rather than assuming that the same module will behave similarly across all cellular backbones.

Overall, the next phase of genome-engineered cellular therapy should avoid equating engineering complexity with therapeutic superiority. Progress will depend on linking each programmable element to a defined resistance mechanism, a clinically measurable biomarker, and a safety profile that can be validated prospectively. For high-risk hematological malignancies, the most useful future products are unlikely to be the most complex by design, but those in which antigen targeting, editing strategy, cellular platform, and monitoring plan are rationally matched to the dominant mode of treatment resistance.

## 9 Conclusion

Genome engineering has changed the way resistance to cellular therapy is understood and addressed in hematological malignancies (Roybal and Lim, 2017). Functional CRISPR screening, single-cell perturbation approaches, and advanced editing technologies have shown that failure after CAR-T or CAR-NK therapy is rarely driven by a single mechanism. Instead, relapse usually reflects interacting resistance modules, including antigen heterogeneity, impaired interferon or antigen-presentation signaling, checkpoint-mediated suppression, myeloid and metabolic constraints, and clonal selection under immune pressure. These findings support a shift from potency-focused cellular engineering toward strategies that are informed by the dominant mechanisms of immune escape and tumor microenvironment-mediated resistance (Lawson et al., 2020).

Next-generation CAR-T and CAR-NK therapies should therefore be designed with resistance biology in mind. Multi-target or logic-gated receptors may be most relevant for antigen-heterogeneous disease, whereas checkpoint-rewired, cytokine-supported, metabolically reinforced, or armored products may be better suited to suppressive immune niches. CAR-NK cells, universal gene-edited products, and stem-cell-derived platforms add further flexibility, particularly when autologous T-cell fitness is poor or repeat dosing is needed. However, these platforms should not be viewed as universally superior alternatives; their value will depend on whether their biological advantages match the resistance features of a specific disease setting.

For high-risk hematologic malignancies, the most useful framework may be to define risk not only by clinical stage, cytogenetics, or prior treatment exposure, but also by resistance architecture. Integrating multi-omics profiling, functional genomic annotation, antigen assessment, and microenvironmental evaluation could help guide the selection of CAR design, editing modules, and cellular platforms. At the same time, many proposed strategies remain early in development and require prospective validation before they can be incorporated into routine clinical decision-making.

Successful translation will also require careful control of genomic safety, delivery specificity, manufacturing consistency, functional stability, and long-term monitoring. As engineered products become more complex, the field should avoid equating the number of edits with therapeutic superiority. The next stage of genome-engineered cellular therapy will depend on linking each engineering choice to a measurable resistance mechanism, a feasible biomarker, and a clinically testable benefit in defined high-risk patient populations.
